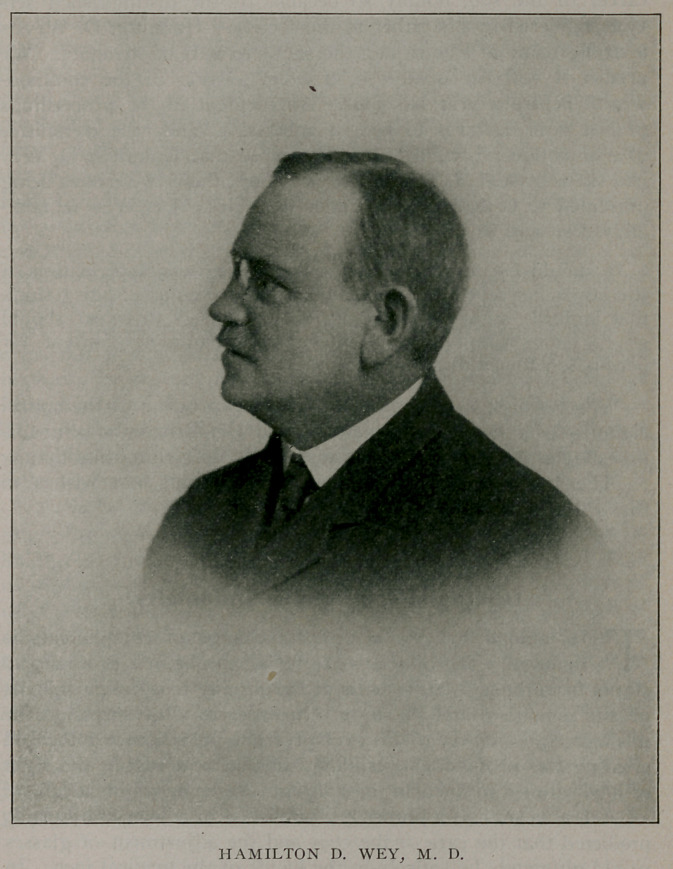# The Ninety-Eighth Annual Meeting

**Published:** 1904-03

**Authors:** 


### BUFFALO MEDICAL JOURNAL.

A Monthly Review of Medicine and Surgery.

*EDITOR:*

WILLIAM WARREN POTTER, M. D.

All communications, whether of a literary or business nature, books for review and exchanges should be addressed to the editor: 284 FRANKLIN STREET, BUFFALO, N. Y.

VOL. XLIII.—LIX. MARCH, 1904. No. 8.

### The Ninety-Eighth Annual Meeting.

THE Medical Society of the State of New York met for the ninety-eighth time in annual session at Albany, January 26-28, 1904. It was presided over by Dr. A. T. Bristow of Brooklyn, who proved a dignified and conscientious chief, who will be long remembered for his courtly bearing in the chair and able conduct of the office of president. While these meetings are always pleasant reunions of the medical profession of the state and instructive in a scientific sense, this one was of special interest from both these viewpoints, and from the additional fact that the committee of conference was expected to make its report on the consolidation of the two state medical bodies,—the Society and the Association.

The committee of the Society, through its chairman, Dr. Elsner, made its report, which consisted of an agreement made with the committee of the Association, of the act passed by the legislature granting permission to the two bodies to consolidate, and of the proposed constitution and by-laws of the Medical Society of the State of New York to be in force after the merger. This report, consisting of about thirty duodecimo pages, had been printed and distributed to the members, but it was necessary to read it in full even though it took considerable time, for such an important proceeding could not be dealt with in a perfunctory manner. Immediately upon the conclusion of the reading by Professor Elsner, Dr. Abraham Jacobi, chairman of the "Committee of Ten," offered the following resolution:

*Resolved*, That the report of the joint committee of conference be accepted, and that the proposed agreement for the consolidation of the Medical Society of the State of New York and the New York State Medical Association be, and the same is, hereby approved; and the president of the Society is hereby authorised and directed to execute the same, in the name and behalf of the Society, and the secretary is hereby authorised and directed to affix the corporation seal thereto; and, be it further

*Resolved*, That the committee of the Society heretofore appointed for the purpose of bringing about the consolidation,—namely, Dr. Henry L. Elsner, Dr. A. Jacobi, Dr. A. Vander Veer, Dr. George Ryerson Fowler, and Dr. Frank Van Fleet be, and they are hereby, continued as such committee, with full power and authority to do whatever may be necessary to carry the agreement into effect.

Dr. D. B. St. John Roosa then obtained the floor and in a brief but eloquent speech, full of meaning sentences and in remarkable keeping with the occasion of goodfellowship which prevailed, seconded the motion to adopt the resolution. Dr. Roosa, in the course of his remarks, called attention, "lest we forget," to the acknowledged fact that, except for the action of the Society in 1882, it had not been possible to obtain legislation establishing state examinations for license to practise, similar action having been hastened in other states as a result of the New York law. He also invited attention to the fact that the Congress of American Physicians and Surgeons was organised as a direct result of the refusal by the American Medical Association to receive the delegates of the Medical Society of the State of New York at Saint Paul in 1882,—an unenlightened policy which had prevailed since that time. The existence of the congress had given the medical profession of New York representation in a national medical body of acknowledged worth and influence which, at least, compensated for the loss of representation in the American Medical Association. In concluding, he said the time had now come to sink all differences of opinion on the questions that had divided the profession for twenty-two years,—a condition made possible by the action of the American Medical Association in shelving the code of ethics at New Orleans last May,—and he hoped the report of the committee would receive the unanimous approval of the Society.

Dr. William Warren Potter, of Buffalo, as a member who, in 1882, had voted to abrogate the code of ethics of the American Medical Association, and as an officer in whom the society had reposed an especial trust, desired both for himself and his colleagues on the examining board to second the motion of Dr. Jacobi, and he added that the thanks of the society were due to this committee that had worked so long, so faithfully, and so indefatigably to bring about unification.

Dr. Robert F. Weir, of New York, also as a member that in 1882, had acted with Drs. Roosa, Potter and others on the question then at issue, seconded the motion before the house.

Dr. Willis G. Macdonald, of Albany, said that though he had joined the society at a period much later than the gentlemen who had previously spoken, he yet sympathised with them in their contentions of 1882; but now he felt that the time had arrived for the profession to reunite, and therefore he heartily seconded the motion. Dr. Roswell Park, of Buffalo, said he belonged to the class of members mentioned by Dr Macdonald and desired to join in seconding the pending motion.

The vote was then taken and was declared by the chair unanimously in the affirmative. The cheering was prolonged and the expressions of good-will were vociferous. After order was restored, Dr DeLancey Rochester, of Buffalo, moved the thanks of the society to the committee of conference, which was also carried by unanimous vote. Thus ended the proceedings relating to this long discussed subject, after another important step toward uniting the society and association had been taken.

---

The scientific program was of special interest and occupied the society for the next two days and a half. The president's address, Professor Hadley's oration and the several symposiums announced, were leading features of a most interesting meeting. The weekly journals all have given full reports of these several topics and of other parts of the program, hence we need not occupy space with unnecessary repetition.

---

At the banquet given Wednesday evening at The Ten Eyck, more than three hundred members and guests spent four hours of unalloyed pleasure. The menu was excellent, the dinner well served, the speeches entertaining and the music inspiriting. President Thornton of the Association and many of the Fellows of that body lent their presence to the joyous occasion which will long me remembered. Dr. Herman Bendell officiated as toastmaster in the most felicitous manner.

---

The election of officers for the ensuing year resulted as follows: president, Hamilton D. Wey, Elmira; vice-president Joseph D. Bryant, New York; secretary, Frederic C. Curtis, Albany; treasurer, O. D. Ball, Albany. The heads of standing committees were chosen as follows: arrangements, Herman Bendell, Albany; by-laws, H. B. Delatour, Brooklyn; hygiene, John L. Heffron, Syracuse; ethics, Edward S. Willard, Watertown; prize essays, A. Jacobi, New York; publication, F. C. Curtis, Albany; legislation, Frank Van Fleet, New York.

---

Dr. Hamilton D. Wey, the newly-elected president is one of the leading physicians in the Southern Tier, is about 50 years

HAMILTON D. WEY, M. D.

of age and has been a permanent member of the society since 1894. He has been for several years chairman of the committee on by-laws and is thoroughly versed in the traditions, methods and interests of the society, all of which will be effectively safeguarded by his administrative policy. His father, Dr. William

C. Wey, was president of the society in 1871, prominent in its councils for thirty years, and was president of the state board of medical examiners for an extended period. Never before in the history of the society have father and son served in its highest office. It should be,—no doubt is,—a proud incident in the career of the son, doubly so because of the distinguished services rendered by his father to the society. Hamilton D. Wey is a strong name in Elmira and the southern part of the state. We predict it will be equally so in every section of the commonwealth before a year has past. An incident in the proceedings of last year deserves to be remembered. When the committee of nomination reported it was discovered that Hamilton D. Wey had six votes and A. T. Bristow two votes, these two names being presented to be balloted for under the rules. Dr. Wey immediately rose and said:

I should be ungrateful if I failed to express appreciation of the honor conveyed by this action of the committee, but I think that logically and geographically the presidency this year should go to Kings county. I, therefore, ask unanimous consent for permission to withdraw my name.

The permission was granted with an expression of the appreciation of his reason and motives, and Dr. Bristow was unanimously elected. Only a strong man could do a thing like that.

The JOURNAL offers its congratulations and best wishes to the distinguished president-elect.

## Figures and Tables

**Figure f1:**